# The Treatment of Pott’s Disease of the Spine

**Published:** 1893-10

**Authors:** A. B. Judson

**Affiliations:** Orthopedic Surgeon to the Out-Patient Department of the New York Hospital; 25 Madison Avenue


### THE TREATMENT OF POTT'S DISEASE OF THE SPINE.

BY A. B. JUDSON, M. D.,

Orthopedic Surgeon to the Out-Patient Department of the New York Hospital.

WHILE caries of any part of the vertebral column cannot be considered an unimportant affection, it is well to recognize the fact that much depends on the region of the spine involved. In the middle dorsal region it is, perhaps, the most serious trouble, excepting malignant disease that can attack the bones of the growing child. In this part of the spinal column the destruction is often extreme and the deformity great, evidently because the affected bones are at the greatest disadvantage mechanically. Lower down, the vertebral bodies are so large that they do not lose their relation of mutual support until the loss of substance is very extensive ; and above, the vertebral bodies, though small, have less weight to sustain. But, in the intermediate portion, not only do the bones feel the incessant movements of respiration, but they are also more widely moved in flexion and extension, and in lateral curving with rotation, than in other parts of the column ; and, furthermore, they are exposed in a peculiar manner to the risk of over-strain, from their position in the middle of the column. I think it is in the experience of all of us that in this middle and upper dorsal region Pott's disease continues longest before consolidation takes place.

Here we have a most striking illustration of the fact that the recovery from articular osteitis is postponed by unfavorable mechanical environment. As joints in the upper extremity, free from the mechanical stress attending locomotion, recover easily, while those which, in the lower extremity, bear the heat and burden of the day, recover only after prolonged and extensive destruction, so articular osteitis, in the cervical region of the spine, is easily curable, while in the upper and middle dorsal region, relief and repair come only after desperate and prolonged risk.

1. Presented at the Pan-American Medical Congress, at Washington, September, 1893.

How can we best assist Nature to cure this disease in this difficult part of the skeleton? The same general rules apply here as in the treatment of articular osteitis in the lower extremities. We cannot cut short the disease by an operation, or by any procedure whatever, but can expect, with confidence, and must promote by our best endeavors, the arrest of destruction and the beginning of repair. What then can we do to put the affected vertebræ in their best attitude, and to raise the defensive and reparative powers of the system to their highest efficiency? As in articular osteitis, occurring elsewhere, we desire (1) to relieve the bone of the duty of supporting weight and concussion; and (2) to prevent the affected joint from motion, believing that the arrest of these two functions, weight-bearing and motion, are essential to good treatment. It does not seem wise to keep the patient recumbent for the long period necessary. In the management of hipdisease, we put the affected limb to bed, so to speak, while the patient is up and about. But a similar resort in Pott's disease is impossible. Since the patient must be up, and, to a certain extent, active in locomotion, our best resort, in my opinion, is to take what benefit can be had from the application of a lever making pressure from behind forwards, in the neighborhood of the posterior projection, and counter-pressure from before backwards at two points, one above and the other below the level of the seat of the disease. In a limited sense, this application relieves the diseased joints from the weight of the body while the patient is up and about, because antero-posterior pressure, thus applied, transfers a part of the weight and concussion incident to standing and walking, from the diseased bodies of the vertebræ to the processes, which remain sound. Having thus (1) removed, so far as is practicable, injurious pressure from the diseased structures, it is obvious that we have also applied the most effective kind of retentive splint for (2) the arrest of motion in the affected joints.

It does not take much practical experience to convince one that efficient pressure, applied in this manner, is productive of good. It may not at once arrest morbid action and induce cicatrization of the carious bone. For these events we must wait for the natural reaction, but it is not difficult to believe that Nature will the more promptly intervene with reparative efforts if our mechanical applications relieve distress and substitute a feeling of strength for weakness and apprehension. A well-applied support at once gives a degree of relief, which finds plain expression in the face and attitude of the patient. As a matter of fact, a feeling of security and comfort is afforded by the use of a corset made from any of the materials in ordinary use. I will not indicate the defects of apparatus of this kind. The inexpensiveness of jackets, and the ease with which they can be obtained and applied, make them of the greatest service to a vast number of patients who otherwise would have no mechanical support whatever. But, when and where it can be done, it is necessary to give the patient the benefit of accurately adjusted antero-posterior pressure.

At the best, antero-posterior pressure, no matter how carefully applied, fails to give all the support which is desirable. This is because the leverage is deficient. In the vertebral column there is found no long bony lever, such as is at hand in making a mechanical application for fixing the knee. There is, rather, a succession of irregular bones, movable upon each other, which, from the nature of the case, impair the success of any attempt to arrest motion or support the column by pressure from behind forwards and counter-pressure from before backwards, because the pressure from before backwards will, a part of it at least, be expended in bending backward portions of the vertebral column above and below the projection. The force thus employed is, however, by no means wasted, as it secures an ultimate improvement in the shape of the trunk, which is often characteristic of patients who have been thus treated.

The apparatus needed is essentially simple, consisting of two parallel uprights united below by a pelvic band, and diverging at their upper ends at the base of the neck, and curving over the tops of the shoulders. Pressure from behind forward is made by two pads attached to the uprights at the level of the projection, and applied a short distance from the median line on each side. Counter-pressure from before backward is made below by a strap passing from one end of the pelvic band to the other in front of the pelvis, and above by straps, one on each side, passing from the upper end of the upright through the axilla, to be buckled to the upright. The most important feature of a brace constructed to carry out these views is the use of mild steel for all the metal parts. The use of this material puts in the hand of the surgeon the power to modify the degree and direction of pressure to the changing shape, and to meet the increasing tolerance of the skin to pressure. The reaction of the skin should receive special and constant attention, and gentle and gradually increasing pressure should be made till the limit of comfortable tolerance is reached.

By patient attention to details, apparatus thus designed may with certainty be made comfortable and efficient. The diffused support furnished by a jacket is often secured by the addition to the simple lever, described above, of aprons and other pieces, which add to the feeling of stability and security without interfering with the chief function of the apparatus, which is to make antero-posterior pressure. One hardly knows where to begin and where to end in the consideration of the details which demand attention in practice of this kind. I will close by saying that cheapness and cleanliness may be promoted by leaving the steel parts of this brace unpolished, and covering them with a single layer of adhesive plaster, and then with strips of canton flannel, or silk, cut bias, and renewed without much trouble as often as may be desired.

25 MADISON AVENUE.

